# Mice infected with *Mycobacterium tuberculosis* are resistant to acute disease caused by secondary infection with SARS-CoV-2

**DOI:** 10.1371/journal.ppat.1010093

**Published:** 2022-03-24

**Authors:** Oscar Rosas Mejia, Erin S. Gloag, Jianying Li, Marisa Ruane-Foster, Tiffany A. Claeys, Daniela Farkas, Shu-Hua Wang, Laszlo Farkas, Gang Xin, Richard T. Robinson

**Affiliations:** 1 Department of Microbial Infection and Immunity; 2 Pelotonia Institute for Immuno-Oncology; 3 Department of Internal Medicine, Division of Pulmonary, Critical Care and Sleep Medicine, Davis Heart and Lung Research Institute; 4 Department of Infectious Disease, The Ohio State University, Columbus, Ohio, United States of America; New Jersey Medical School, UNITED STATES

## Abstract

*Mycobacterium tuberculosis* (Mtb) and SARS-CoV-2 (CoV2) are the leading causes of death due to infectious disease. Although Mtb and CoV2 both cause serious and sometimes fatal respiratory infections, the effect of Mtb infection and its associated immune response on secondary infection with CoV2 is unknown. To address this question we applied two mouse models of COVID19, using mice which were chronically infected with Mtb. In both model systems, Mtb-infected mice were resistant to the pathological consequences of secondary CoV2 infection, and CoV2 infection did not affect Mtb burdens. Single cell RNA sequencing of coinfected and monoinfected lungs demonstrated the resistance of Mtb-infected mice is associated with expansion of T and B cell subsets upon viral challenge. Collectively, these data demonstrate that Mtb infection conditions the lung environment in a manner that is not conducive to CoV2 survival.

## Author summary

*Mycobacterium tuberculosis* (Mtb) and SARS-CoV-2 (CoV2) are distinct organisms which both cause lung disease. We report the surprising observation that Mtb-infected mice are resistant to secondary infection with CoV2, with no impact on Mtb burden and resistance associating with lung T and B cell expansion.

## Introduction

The world is currently in the midst of two lung disease pandemics: COVID19 and tuberculosis (TB), the causative agents of which are SARS-CoV-2 (CoV2) and *Mycobacterium tuberculosis* (Mtb), respectively. Although COVID19 and TB both pose enormous health challenges, especially in countries where COVID19 vaccines are scarce, it unknown what if any effect Mtb infection has on host responses to CoV2 as there are few clinical reports of Mtb/CoV2 coinfection in the absence of other comorbidities [1,2]. On the one hand, CoV2 infection may exacerbate the inflammatory response and pulmonary complications experienced by individuals with TB [3], analogous to that which is observed in the Mtb/Influenza A or Mtb/CMV coinfected individuals [4–7]. On the other hand, there is an inverse relationship between TB incidence rates and COVID19 mortality in numerous countries [8], and *Mycobacterium* spp express several proteins homologous to CoV2 antigens [9–11], raising the possibility that adaptive immune responses to Mtb may confer heterologous immunity against CoV2. To definitively address whether Mtb-infection impacts CoV2 elicited lung disease in a controlled setting, we applied two mouse models of COVID19 (CoV2 infection of K18-hACE2 mice [12], and mouse-adapted CoV2 [MACoV2] infection of C57BL/6 mice [13]), using mice that were chronically infected with Mtb. The results below support a model wherein Mtb infection confers resistance to secondary infection with CoV2 and its pathological consequences. The implications of these data for our understanding of COVID19 susceptibility and the limitations of our study are discussed.

## Results

**Fig 1:**
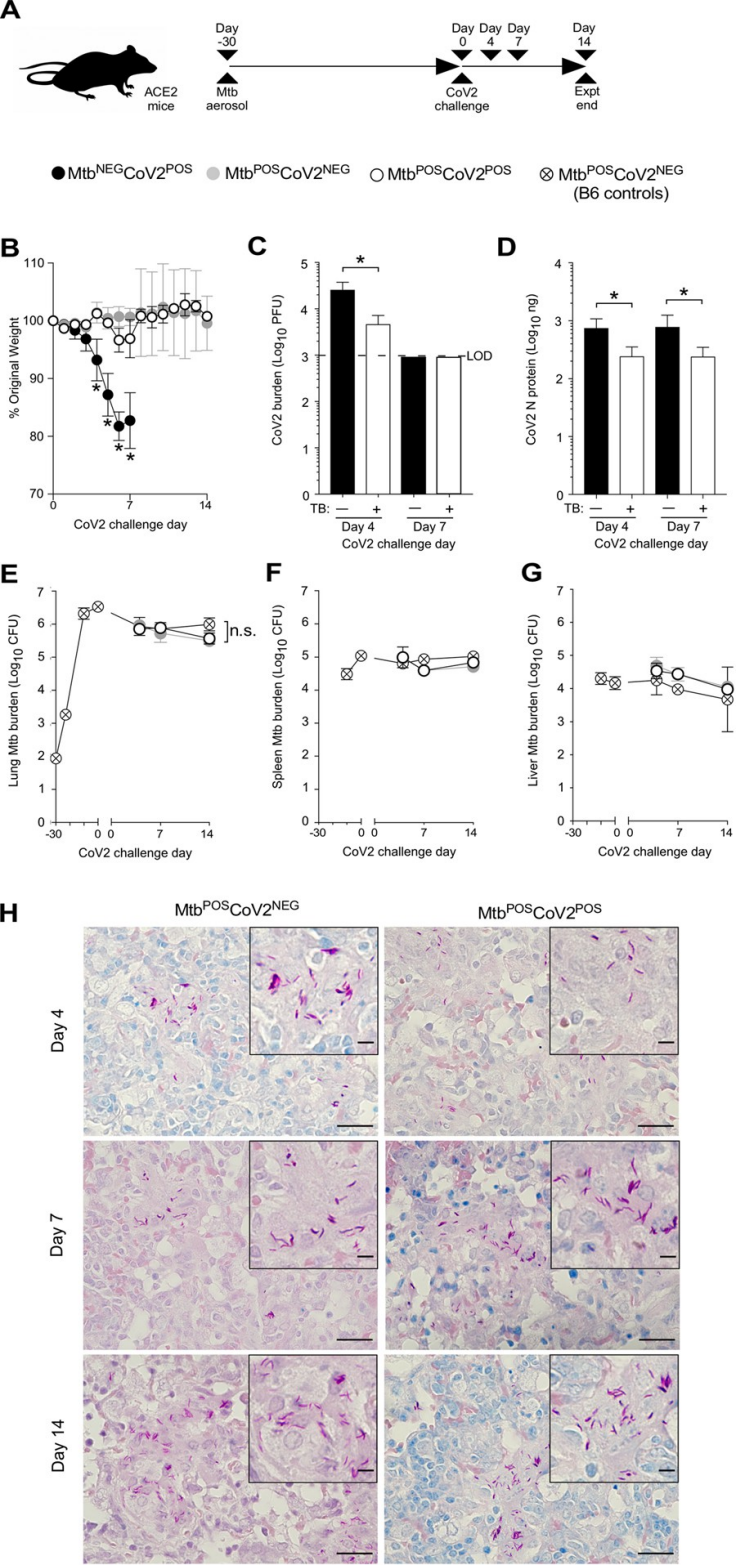
Mtb-infected ACE2 mice are resistant to secondary infection with CoV2. (**A**) Experimental overview of our ACE2:CoV2 model studies, wherein mice were infected via aerosol with Mtb (Day -30) and challenged 30 days later (Day 0) with CoV2. On post-challenge Day 4, Day 7 and Day 14, tissues were collected for microbiological and immunological assessments. Experimental groups included ACE2 mice which were not infected with Mtb prior to CoV2 challenge (Mtb^NEG^CoV2^POS^), ACE2 mice which were infected with Mtb but challenged with sterile saline (Mtb^POS^CoV2^NEG^), ACE2 mice which were infected with Mtb prior to CoV2 challenge (Mtb^POS^CoV2^POS^), and B6 controls which were infected with Mtb (to determine what if any impact human ACE2 transgene expression alone has on Mtb burdens). (**B**) The percent weight change experienced by each group of ACE2 mice following CoV2 challenge, as normalized to the original weight of each mouse. (**C**) CoV2 PFU burdens and (**D**) CoV2 N protein levels in the lungs of Mtb^NEG^CoV2^POS^ and Mtb^NEG^CoV2^POS^ mice. (**E-G**) Mtb CFU burdens in the (**E**) lungs, (**F**) spleen and (**G**) liver of Mtb^POS^CoV2^NEG^ and Mtb^POS^CoV2^POS^ mice, as well as B6 controls throughout the experiment time course. In each graph the following legend applies: Mtb^NEG^CoV2^POS^, black circles or bars; Mtb^POS^CoV2^NEG^, gray circles; Mtb^POS^CoV2^POS^, white circles or bars. (**H**) Representative micrographs of AFB stained lung sections, as collected from Mtb^POS^CoV2^NEG^ and Mtb^POS^CoV2^POS^ mice at the indicated times post-challenge. In each micrograph, the large scale bar is 20 μM and inset scale bar is 5 μm. This experiment was repeated twice, each with similar results (4 mice/group/timepoint). *, p ≤ 0.05 as determined by either Student’s t-test or ANOVA; n.s., not significant.

Details regarding the origin, culture, preparation and authentication of CoV2 (strain USA-WA1/2020), MACoV2 (strain MA10) and Mtb (strain H37Rv) are provided in our *Materials & Methods*. To determine if host responses to CoV2 are affected by Mtb-infection, K18-hACE2 (ACE2) and C57BL/6 (B6) mice were infected with low dose Mtb (~90 CFU) via aerosol delivery; thirty days later, the ACE2 mice were challenged with CoV2 (~25K PFU) via intranasal delivery (**Fig 1A**). These Mtb/CoV2 co-infected (Mtb^POS^CoV2^POS^) ACE2 mice were monitored daily for changes in weight, as were two control groups: ACE2 mice which were Mtb-infected at the same time (Day -30) but challenged with sterile media (Mtb^POS^CoV2^NEG^), and ACE2 mice which were not Mtb-infected prior to CoV2 challenge (Mtb^NEG^CoV2^POS^). On post-challenge Days 4, 7 and 14, groups of mice were euthanized and the lungs and other tissues were removed to assess Mtb and CoV2 burdens, as well as a number of immunological readouts. All mice were identically housed for the duration of the entire experiment. As anticipated, Mtb^NEG^CoV2^POS^ ACE2 mice lost a significant portion of body weight by post-challenge Day 7 (≤20%) (**Fig 1B**). Mtb^POS^CoV2^POS^ ACE2 mice, however, did not lose significant body weight and were otherwise indistinguishable from Mtb^POS^CoV2^NEG^ controls (**Fig 1B**). On post-challenge Day 4, lung CoV2 burdens were lower in Mtb^POS^CoV2^POS^ mice relative to Mtb^NEG^CoV2^POS^ mice, as assessed by either plaque assay (**Fig 1C**) or CoV2 N protein measurement (**Fig 1D**). Challenge with CoV2 did not affect Mtb growth, as Mtb CFU burdens in Mtb^POS^CoV2^POS^ and Mtb^POS^CoV2^NEG^ lungs did not differ after challenge (**Fig 1E**), nor did they differ in spleen (**Fig 1F**) or liver (**Fig 1G**). Consistent with the above Mtb CFU results, the abundance of acid fast bacilli (AFB) was also similar between Mtb^POS^CoV2^POS^ and Mtb^POS^CoV2^NEG^ lungs (**Fig 1H**). Transgenic human ACE2 expression also does not affect Mtb growth, as CFU burdens in Mtb^POS^CoV2^NEG^ ACE2 mice were indistinguishable from Mtb-infected B6 controls (**Fig 1E, 1F and 1G**).

**Fig 2:**
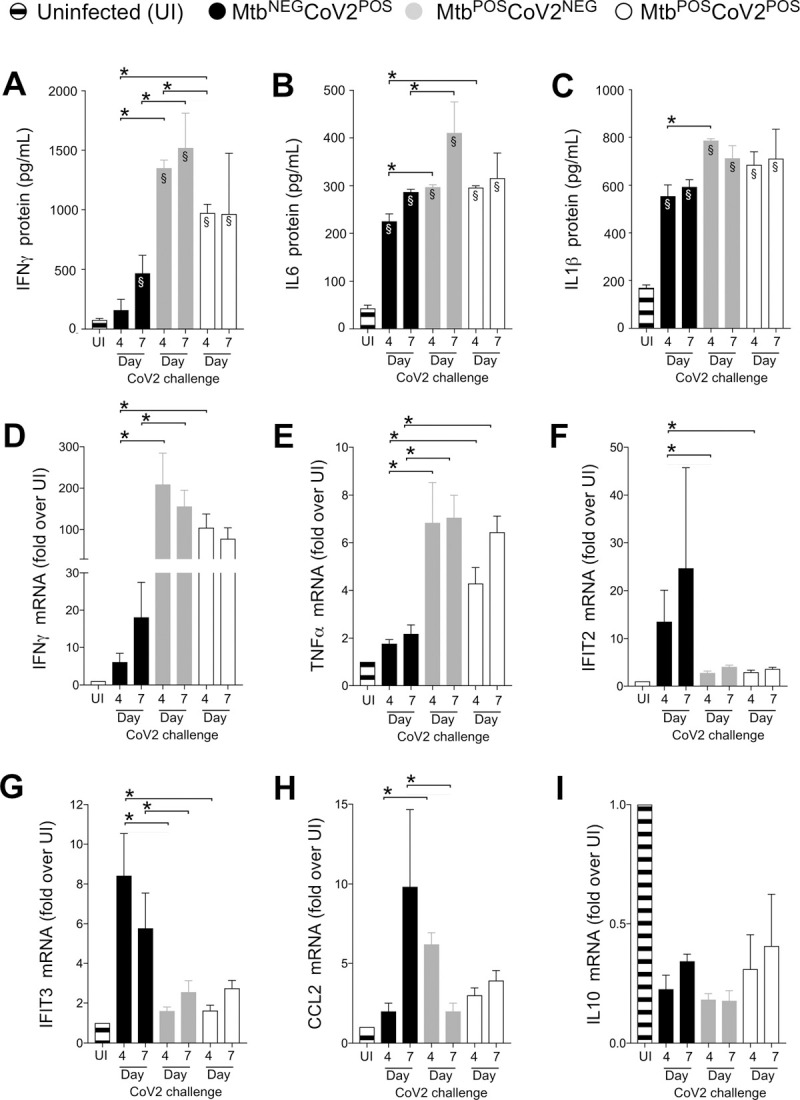
CoV2-elicted cytokine responses are muted in the presence of Mtb infection. On the indicated days, lung tissue from Mtb^NEG^CoV2^POS^, Mtb^POS^CoV2^NEG^, Mtb^POS^CoV2^POS^ and uninfected (UI) ACE2 mice was used to measure (**A-C**) protein levels of (**A**) IFNγ, (**B**) IL6 and (**C**) IL1β, as well as (**D-I**) mRNA levels of (**D**) IFNγ, (**E**) TNFα, (**F**) IFIT2, (**G**) IFIT3, (**H**) CCL2 and (**I**) IL10. This experiment was repeated twice, each with similar results (4 mice/group/timepoint). *, p ≤ 0.05 as determined by either Student’s t-test or ANOVA; §, significant relative to UI protein levels.

We next assessed the impact of Mtb infection on CoV2-elicited immune responses in the lung, using tissue from the same ACE2 transgenic mice described above. CoV2 infection elicits the expression of multiple inflammatory genes in mouse lungs [12]. Consistent with this, protein levels of IFNγ, IL6 and IL1β were elevated in Mtb^NEG^CoV2^POS^ lungs post-challenge Day 4 and/or Day 7, relative to uninfected (UI) controls (**Fig 2A, 2B and 2C**). In Mtb^POS^CoV2^NEG^ mice, lung protein levels of IFNγ, IL6 and IL1β were even higher, and were not affected by CoV2 challenge (**Fig 2A, 2B and 2C**, compare Mtb^POS^CoV2^NEG^ and Mtb^POS^CoV2^POS^ levels). This pattern, wherein Mtb monoinfection induces high levels of a gene expression that are unchanged upon CoV2 challenge, was also observed for IFNγ and TNFα at the mRNA level (**Fig 2D and 2E**); however and notably, the resistance of Mtb^POS^CoV2^POS^ mice did not associate with elevated expression of the antiviral genes IFIT2 and IFIT3, which are otherwise induced in Mtb^NEG^CoV2^POS^ mice (**Fig 2F and 2G**), nor was CoV2 able to induce CCL2 expression in the presence of Mtb (**Fig 2H**). Expression of the anti-inflammatory cytokine IL10 was low in all experimental groups relative to UI controls (**Fig 2I**).

**Fig 3:**
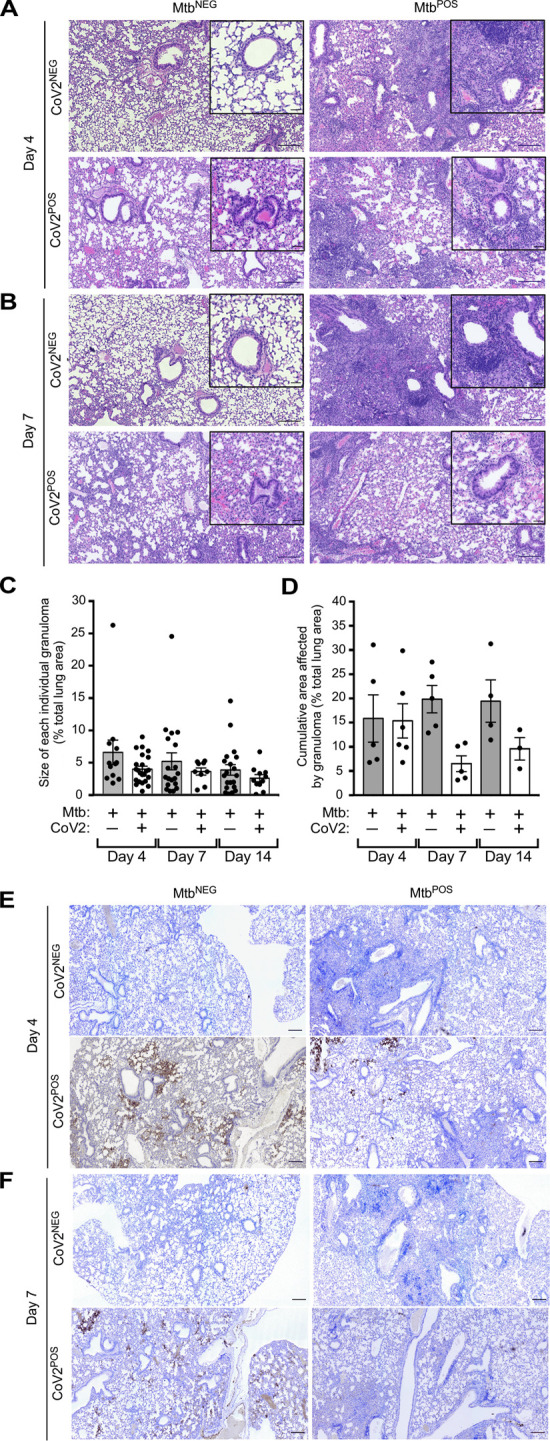
CoV2 infection of the airways and associated pneumonia are attenuated in the presence of Mtb. **(A-B)** Representative micrographs of H&E stained lung sections, sections from each experimental group, as collected on (**A**) Day 4 or (**B**) Day 7 post challenge. (**C-D**) Quantitative analysis of H&E stains demonstrating (**C**) the size of each individual TB granuloma relative to the total lung area, with each dot representing an individual TB granuloma on the indicated Days, as well as (**D**) the cumulative area taken up by all granulomas in an individual mouse lung, with each dot representing an individual mouse on the indicated Days. Bars represent the mean +/- SD. **(E-F)** Representative micrographs of CoV2 N protein IHC stained lung sections from each experimental group, as collected on (**E**) Day 4 or (**F**) Day 7 post challenge. In each micrograph the large scale bar represents 200 microns; insets are 50 microns.

At a histological level, the lungs of Mtb^NEG^CoV2^POS^ mice exhibited a number of previously reported features [14] by post-challenge Day 4 (**Fig 3A**) and Day 7 (**Fig 3B**), including diffuse alveolar damage with inflammatory infiltrates and alveolar necrosis. Since these features were also observed in granulomatous lesions of Mtb^POS^CoV2^NEG^ lungs, a hallmark of Mtb infection, we could not use histology to observe whether Mtb inhibits CoV2-induced inflammation and alveolar necrosis. What could be observed, however, were differences between Mtb^NEG^CoV2^POS^ and Mtb^POS^CoV2^POS^ lungs with regards to hyaline membrane formation and pneumonia in the terminal bronchioles by Day 7 (**Fig 3B inset**), which were notable in Mtb^NEG^CoV2^POS^ lungs but absent from Mtb^POS^CoV2^POS^ lungs (pneumonia is not typical of Mtb-infected mice on the B6 background until ~1 year after infection [15]). The sizes of each individual TB granuloma in Mtb^POS^CoV2^NEG^ and Mtb^POS^CoV2^POS^ mice were similar at all time points (**Fig 3C**), as were the cumulative lung area taken by all TB granulomas (**Fig 3D**). Consistent with our assessment of lung CoV2 burdens (**Fig 1C and 1D**), anti-N protein immunohistochemistry (IHC) staining demonstrated fewer and less intense IHC+ regions within Mtb^POS^CoV2^POS^ lungs, relative to Mtb^NEG^CoV2^POS^ lungs (**Fig 3E and 3F**). Notably, the few IHC+ regions which were observed in Mtb^POS^CoV2^POS^ lungs were distal to granulomatous lesions that contain Mtb (**Fig 3E**).

**Fig 4:**
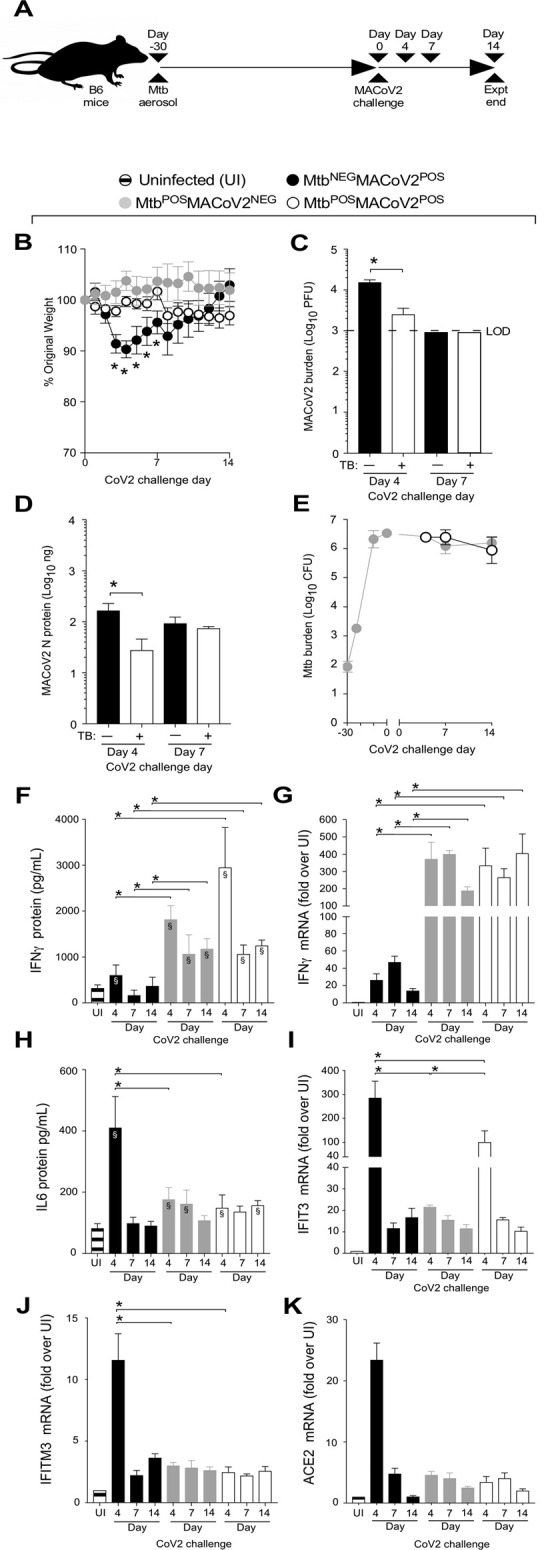
Mtb-infected B6 mice are resistant to secondary infection with MACoV2. (**A**) Experimental overview of our B6:MACoV2 model studies, wherein mice were infected via aerosol with Mtb (Day -30) and challenged 30 days later (Day 0) with MACoV2. On post-challenge Days 4, 7 and14 we collected lung tissue for microbiological and immunological assessments. Experimental groups included B6 mice which were not infected with Mtb prior to MACoV2 challenge (Mtb^NEG^MACoV2^POS^), B6 mice which were infected with Mtb but challenged with sterile saline (Mtb^POS^MACoV2^NEG^), and B6 mice which were infected with Mtb prior to CoV2 challenge (Mtb^POS^MACoV2^POS^). (**B**) The percent weight change experienced by each group of B6 mice following MACoV2 challenge, as normalized to the original weight of each mouse. (**C-D**) Lung viral burdens in Mtb^NEG^MACoV2^POS^ and Mtb^POS^MACoV2^POS^ mice, as measured by (**C**) MACoV2 PFU or (**D**) MACoV2 N protein concentration on the indicated days, as well as (**E**) lung Mtb CFU burdens at the same timepoints. (**F, H**) Lung protein levels of (**F**) IFNγ and (**H**) IL6, as well as (**G, I-K**) mRNA levels of (**G**) IFNγ, (**I**) IFIT3, (**J**) IFITM3 and (**K**) ACE2. This experiment was repeated twice, each with similar results (4 mice/group/timepoint). *, p ≤ 0.05 as determined by either Student’s t-test or ANOVA; §, significant relative to UI protein levels.

To determine whether Mtb-induced resistance to CoV2 was specific to the ACE2 transgenic model of COVID19, we performed the same set of experiments using a second mouse model of COVID19: MACoV2 infection of B6 mice [13]. As before, our experimental groups included B6 mice which were uninfected prior to MACoV2 challenge (Mtb^NEG^MACoV2^POS^), or Mtb-infected 30 days prior to challenge with MACoV2 (Mtb^POS^MACoV2^POS^) or vehicle control (Mtb^POS^MACoV2^NEG^) (**Fig 4A**). Whereas ACE2 mice which lost ≤20% body weight within 7 days of CoV2 challenge (**Fig 1B**), MACoV2 induced weight loss was less dramatic, with Mtb^NEG^MACoV2^POS^ mice losing ≤10% body weight within 7 days of MACoV2 challenge (**Fig 4B**). Nevertheless and consistent with our ACE2 model results, Mtb^POS^MACoV2^POS^ were resistant to MACoV2-elicited weight loss (**Fig 4B**), had lower viral burdens compared to Mtb^NEG^MACoV2^POS^ mice (**Fig 4C and 4D**) and no change in lung Mtb burdens following virus challenge (**Fig 4E**). Following virus challenge, Mtb^NEG^MACoV2^POS^ lungs exhibited transient increases in protein and mRNA levels of IFNγ (**Fig 4F and 4G**), IL6 (**Fig 4H**), IFIT3 (**Fig 4I**), IFITM3 (**Fig 4J**) and ACE2 (**Fig 4K**), consistent with previous reports of CoV2 inducing expression of its own receptor [16]. As was also observed in the ACE2 model (**Fig 2A and 2B**), protein levels of IFNγ and IL6 were already high in Mtb^POS^MACoV2^NEG^ lungs and unaffected by MACoV2 challenge (**Fig 4F and 4H**). MACoV2 elicited IFIT3 expression in both Mtb^NEG^MACoV2^POS^ and Mtb^POS^MACoV2^POS^ lungs, albeit lower in the latter group (**Fig 4J**). The resistance of Mtb-infected B6 mice to CoV2 was not attributable to an absence of lung ACE2 expression, as Mtb^POS^MACoV2^POS^ mice expressed higher than UI levels of ACE2 (**Fig 4K**); unlike Mtb^NEG^MACoV2^POS^ mice, however, ACE2 expression in Mtb^POS^MACoV2^POS^ lungs was not affected by MACoV2 challenge (**Fig 4K**).

**Fig 5:**
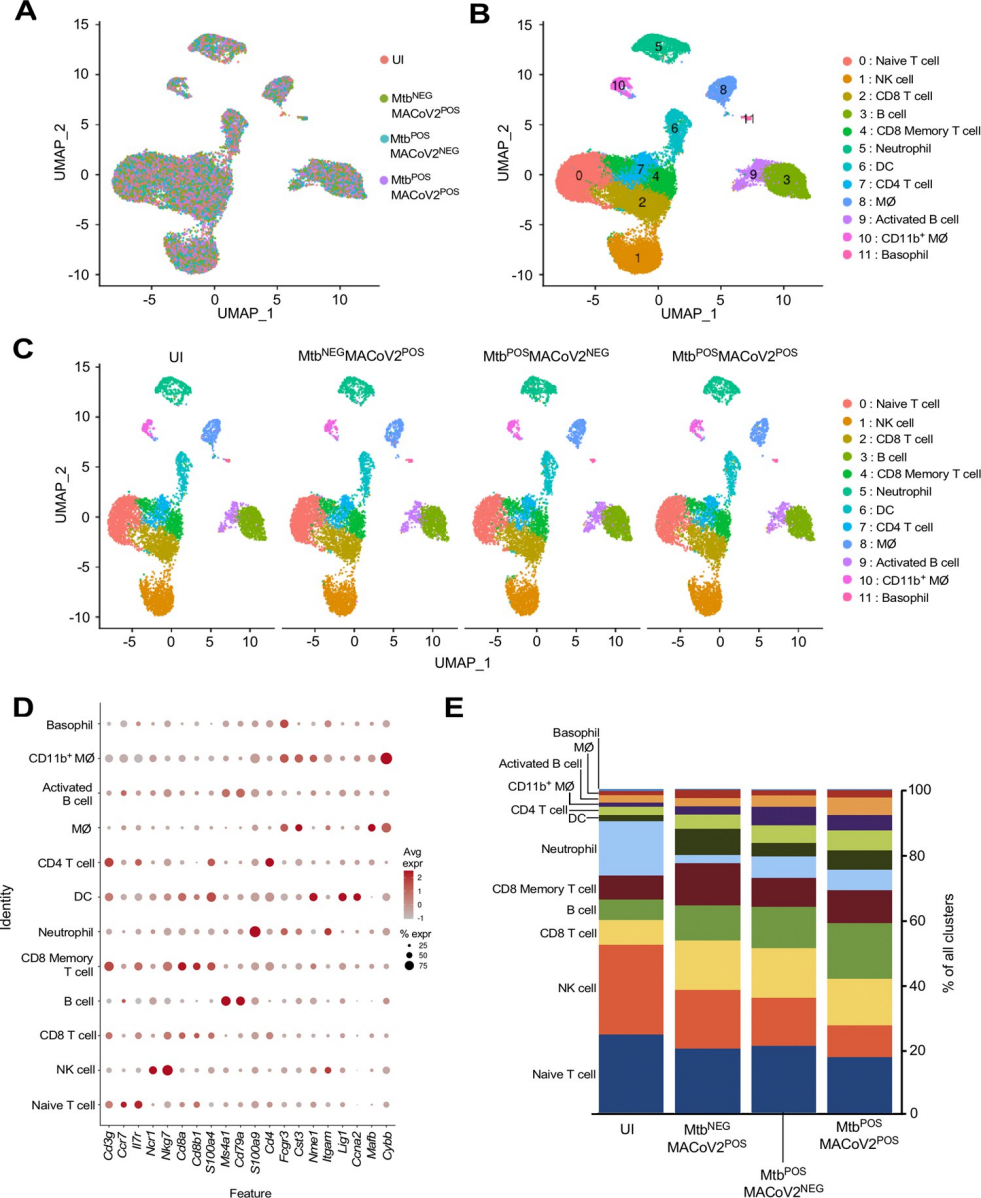
Lung T and B cell subsets expand upon challenge of Mtb^POS^ mice with MACoV2. As an unbiased means to define and compare the lung immune landscape, live CD45+ cells were purified from the lungs of four experimental groups (Uninfected, UI; Mtb^NEG^MACoV2^POS^; Mtb^POS^MACoV2^NEG^; Mtb^POS^MACoV2^POS^) on post-challenge Day 7 and used for scRNA analysis. (**A-C**) t-SNE plots of the resulting data, either (**A-B**) pooled across groups or (**C**) segregated by group to show (**A**) the extent of overlay and (**B-C**) clustering of data into 12 immune lineages. (**D**) The distribution and expression patterns of lineage defining genes which we used to annotate each cluster, as pooled from all group data (individual group data are shown in **S1 Fig**). (**E**) The proportion of each immune lineage in the lungs of each experimental group. MØ, macrophage; DC, dendritic cell; NK, natural killer.

Finally, to discern the lung immune environment associated with MACoV2 resistance in Mtb-infected mice, we used scRNA-seq to analyze live CD45+ cells from the lungs of each group (UI, Mtb^NEG^MACoV2^POS^, Mtb^POS^MACoV2^NEG^ and Mtb^POS^MACoV2^POS^) on post-challenge Day 7. This timepoint enabled us to analyze immune cells after PFU are no longer detectable (**Fig 4C**). Live CD45+ cells were purified from the lungs of each group (4 mice per group) and used to prepare single-cell transcriptome datasets. These datasets separated into 12 clusters using the dimensionality reduction and clustering algorithms in the 10X Cell Ranger pipeline (**Fig 5A, 5B and 5C**). The expression profile of 20 myeloid and lymphoid lineage markers (*S100a4*, *S100a9*, *Cd8b1*, *Cd4*, *Cd79a*, *Ms4a1*, *Cybb*, *Mafb*, *Cd3g*, *Fcgr3*, *Cst3*, *Nme1*, *Itgam*, *Cd8a*, *Lig1*, *Ccna2*, *Ccr7*, *Il7r*, *Ncr1* and *Nkg7*) allowed us to assign biological identities to each cluster (**Fig 5D**). For each lineage marker, the average expression and percent positivity within each cluster were similar across all experimental groups (**S1 Fig)**. We identified four T cell clusters (clusters 0, 2, 4 and 7), two B cell clusters (clusters 3 and 9), three myeloid-cell clusters (clusters 6, 8, and 10), one basophil cluster (cluster 11), one neutrophil cluster (cluster 5), and one natural killer–cell cluster (cluster 1) (**Fig 5A**). The extent to which these clusters were represented among all CD45+ cells varied by group (**Fig 5C and 5E**). We observed that innate clusters (i.e. NK, neutrophil, DC, MØ, CD11b+ MØ and basophils) comprised 50% of the UI lung, with T cells (42%) and B cells (8%) making up the difference (**Fig 5D**). In Mtb^NEG^MACoV2^POS^ lungs, the representation of T cell (51%) and B cell (16%) clusters was higher, as were DC (4%) and MØ (7%) clusters. Relative to UI lungs, the Mtb^POS^MACoV2^NEG^ lung was characterized by the expansion of nearly all immune clusters (CD8 T cells, 8→15%; B cells, 6→13%; CD8 memory T cells, 7→9%; DCs, 2→4%; CD4 T cells, 3→6%; CD11b+ MØ, 1→2%; activated B cells, 2→4%; MØ, 1→5%) at the expense of neutrophils (17→7%), NK cells (28→15%) and naïve T cells (24→21%). Importantly, the profile of Mtb^POS^MACoV2^POS^ lungs closely resembled that of Mtb^POS^MACoV2^NEG^ lung, with the exception of expanded B cell (17%), CD8 memory T cell (10%), DC (6%) and activated B cell (5%) clusters, again at the expense of neutrophils, NK cells and naïve T cells (**Fig 5D**). Collectively, our scRNA seq data demonstrates the resistance of Mtb-infected mice to MACoV2 is associated with a lung immune environment that is largely similar to that observed in Mtb monoinfected lungs, with the exception of expanded T and B cell clusters.

## Discussion

Our results demonstrate that Mtb infected mice on the C57BL/6 genetic background are resistant to secondary infection with CoV2 and its pathological consequences. With regards to the mechanism of resistance, we believe the inflammatory nature of Mtb infection creates a lung environment that is inhospitable to CoV2 propagation. In the absence of Mtb, CoV2 enter cells via ACE2, propagates and triggers and inflammatory response that extends after CoV2 clearance and causes declines in lung function and death [17]. In the presence of Mtb, CoV2 entry is likely unaffected since ACE2 is abundantly expressed in the Mtb-infected lung, but the extent of CoV2 propagation is low and the immunopathological responses typically triggered in mice (i.e. weight loss, pneumonia) are muted. This is likely due to one or both of the following reasons: (1) Mtb infected lungs already contain an array of immune innate lineages which restrict CoV2, or (2) Mtb elicits an adaptive immune response that cross reacts with CoV2 antigens and offers heterologous immunity. This latter explanation is supported by recent epidemiological studies of COVID among individuals vaccinated with *M*. *bovis* BCG [18–20], which depending on the strain has significant antigenic overlap with Mtb [21,22]. The limitations of our study include its being performed in mice, which of course do not recapitulate all aspects of TB or COVID in humans, nor have we examined the long term impact of CoV2 on the host response to Mtb as we terminated our study fourteen days after CoV2 challenge. That said, we believe animal models of TB and COVID are ideal for studies of this nature because—if studies of COVID in individuals with other chronic lung diseases are any guide [23,24]—it will likely be difficult to tease apart the impact of TB on COVID outcomes in humans given that individuals with TB often have numerous other comorbidities (e.g. malnourishment, HIV) that confound interpretation. Translated to human COVID susceptibility, our results suggest that individuals infected with Mtb generate an immune response that offers a degree of protection from subsequent or secondary infection with CoV2.

We believe our study raises a number of important questions which can be answered by future research, including (but not limited to) the following:

*Do our findings extend to other models of TB which better recapitulate the range of human disease*? As expertly reviewed elsewhere [25], mice are an imperfect model of TB because they do not display the spectrum of pathology observed in humans with TB. This is especially true of mice on the C57BL/6 genetic background, which are resistant to TB in the absence of any induced or genetic immunodeficiency [26]. It will be important to test if our results can be generalized to other models which do display a greater range of TB and COVID disease forms (e.g. NHP, Diversity Outbred mice) [27–29]. Related to this is the question of whether the resistance we observed varies with the stage of TB disease, as the cellular composition, cytokine milieu and protective capacity of the TB granuloma waxes and wanes over time [30].

*What is the mechanism of acute COVID disease resistance in Mtb-infected mice*? Whether the expansion of B cell or CD8 T cells we observed in Mtb^POS^CoV2^POS^ mice is indicative of their having protective properties in the context of co-infection can be determined via classic immunology methods such as lineage depletions and/or adoptive transfers. So too can sera from Mtb infected animals be tested for neutralizing or protective capacity against CoV2 challenge, since there may be antibodies present to the numerous mycobacterial proteins that are homologous to CoV2 antigens [9–11].

*How is the human immune response to TB affected by COVID*? As the acute crisis stage of the COVID pandemic has (hopefully) begun to move into the past with the advent of effective vaccines, scattered reports are emerging of interesting associations between COVID and Interferon-Gamma Release assay (IGRA) results. IGRAs are whole-blood tests which detect secretion of IFNγ in response to two or three synthetic, Mtb-specific peptides depending on the manufacturer (QuantiFERON-TB Gold In-Tube test: ESAT-6, CFP-10 & TB7.7; T-SPOT TB test: ESAT-6 & CFP-10), relative to a negative control antigen (nil) and a positive control antigen (the polyclonal mitogen phytohemagglutinin, PHA). A “positive IGRA” is indicative of active or prior Mtb infection; an “indeterminate IGRA” can stem from either an insufficient response to the positive control, or too high a background response to the negative control. Consistent with our present animal model study, Gupta et al observed that a cohort of 20 asymptomatic COVID patients were more likely to have a positive IGRA than 20 severely ill COVID patients [31], suggesting those with prior TB exposure are less likely to develop symptoms following CoV2 infection. Equally interesting are elevated rates of indeterminate IGRA in hospitalized COVID patients, due to too low IFNγ levels in the positive control blood collection tubes [32,33]. Another recent study [34] of 10 COVID patients with active TB and 11 COVID patients with latent TB shows that both groups retain their ability to respond to Mtb antigens as measured via IGRA, but (compared to 63 COVID patients with no history of TB) the blood of those with active TB (not latent TB) are less responsive to CoV2 antigens. Our animal model data would suggest this later result could be due to either the inflammatory environment limiting CoV2 replication (resulting in less viral antigens for generating an adaptive response in the first place). Alternatively since most of the T cell compartment localizes to the lung (away from the circulation) in those with active TB, due to the array of chemokines that attract both Mtb-specific and non-Mtb-specific T cells alike [35], lower CoV2 antigen responsiveness in the blood may reflect lower T cell numbers in the blood. That hospitalized individuals with COVID are often treated with corticosteroids is an additional confounder of human TB/COVID co-infection studies.

Finally, TB and COVID are ongoing pandemics and our results should not be used to support any assertion that those with TB (or history of TB) will not get COVID, nor should they be used to support any assertion that those with TB (or history of TB) should not get vaccinated against COVID. Hospitalization and severe forms of COVID can now be prevented by several vaccines; the US Centers for Disease Control and Prevention, European Centre for Disease Prevention and Control, and World Health Organization each maintain updated lists of these vaccines on their respective websites (https://www.cdc.gov, https://www.ecdc.europa.eu, https://www.who.int). We do hope and anticipate that our results will be used to support future studies of patient populations and animal models to improve knowledge of the TB/COVID interaction.

## Materials & methods

### Ethics statement

All animal studies were reviewed and approved by the local Institutional Animal Care and Use Committee (OSU IACUC) prior to their onset (Approval # 2018A00000076 and 2020A00000044).

### SARS-CoV-2 culture, preparation and authentication

All experiments involving SARS-CoV-2 followed procedures and protocols that are approved by The Ohio State University (OSU) Institutional Biosafety Committee. SARS-CoV-2, isolate USA-WA1/2020, was obtained from Biodefense and Emerging Infections Research Resources Repository (BEI Resources, Batch # 70034262). Mouse adapted SARS-CoV-2 variant strain MA10 [36] was likewise provided by BEI Resources (Cat # NR-55329). Virus was cultured, prepared and authenticated as we recently reported [37]. Namely, to establish the viral stocks used in our studies, a virus aliquot was thawed, diluted 1:10,000 in incomplete DMEM (Gibco; supplemented with 4.5 g/L D-glucose, 110 mg/L sodium pyruvate) and added to confluent VeroE6 cells (ATCC). Cells were incubated with virus for 1h (37°C, 5% CO_2_), after which time the media was replaced with complete DMEM (i.e. DMEM prepared as above, further supplemented with 4% heat-inactivated fetal bovine serum) and the cells were incubated for 3 days (37°C, 5% CO_2_) to allow virus propagation. After that period, visual inspection under light microscopy demonstrated near complete death of the infected VeroE6 cells. The supernatant was collected into 50mL conicals, centrifuged at low speed to remove cell debris and subsequently aliquoted, frozen and stored at -80°C. These frozen aliquots served as the stock tubes for all subsequent experiments. The live virus titer of our frozen aliquots was determined via the plaque assay described below. SARS-CoV-2 stocks were authenticated using a clinically validated clinical next-generation sequencing assay [38].

### *Mycobacterium tuberculosis* culture, preparation and authentication

All experiments involving *M*. *tuberculosis* (Mtb) followed procedures and protocols that are approved by The Ohio State University (OSU) Institutional Biosafety Committee. The virulent Mtb strain H37Rv (Trudeau Institute, Saranac Lake, NY) was grown in Proskauer Beck medium containing 0.05% tyloxapol to mid-log phase (37°C, 5% CO_2_) and frozen in 1-ml aliquots at −80°C. The live bacteria titer of our frozen aliquots was determined via plating serial dilutions on 7H11 agar media. To authenticate our Mtb stock we confirmed that the colony morphology, *in vitro* growth characteristics and *in vivo* virulence were consistent with our previous studies using the H37Rv strain [39].

### Mice

All mice were treated in accordance with OSU Institutional Animal Care and Use Committee (IACUC) guidelines and approved protocols. C57BL/6 and hemizygous K18-hACE C57BL/6J mice (strain: 2B6.Cg-Tg(K18-ACE2)2Prlmn/J) were purchased from Jackson Laboratory (Bar Harbor, ME) and housed at OSU within an AALAC-accredited facility (University Laboratory Animal Resources, ULAR).

### Aerosol Mtb infection

Mice were aerosol infected with Mtb H37Rv per our previous studies using the Glas-Col inhalation system [39]. For bacterial load determinations, the lungs, spleen, and liver were aseptically removed and individually homogenized in sterile normal saline (Gentle Macs system, program “RNA” run 2X). Serial dilutions of each organ were then plated on 7H11 and colonies counted after 2–3 weeks incubation at 37°C 5% CO_2_. Lungs from control mice were plated on post-infection Day 1 to verify the delivery of ~80 Mtb CFU.

### Intranasal CoV2 challenge

Mice that were either uninfected (UI) or previously infected with aerosol Mtb (Mtb^POS^) mice were challenged with either CoV2 or MACoV2. At the time of challenge, mice were anesthetized with isoflurane, weighed and held at a semi-supine position while 50 μL of CoV2-containing PBS (2.5 × 10^4^ PFU) or MACoV2 (2.5 × 10^4^ PFU) was given via intranasal (i.n.) instillation. Control mice were given the same volume of sterile PBS, using the same anesthesia and i.n. instillation protocol. After i.n. instillation, each mouse was returned to its home cage, house and monitored daily for changes in weight or body condition. For viral load determinations, the lungs of challenged animals were aseptically removed and individually homogenized as described above; serial dilutions were then used in the plaque assay described below.

### CoV2 plaque assay

A modified version of the plaque assay developed by the Diamond laboratory [40] was used to determine lung viral burdens in challenged animals, the details of which we have reported [41]. Namely, one day prior to the assay start we seeded 12-well with VeroE6 cells and incubated overnight (37°C 5% CO_2_) such that each well was confluent by the assay start. On the day of the assay, serial dilutions of virus-containing material (e.g. lung homogenate) were prepared in cDMEM and warmed to 37°C. Media from each well of the 12-well plate was gently removed via pipette and replaced with 500uL of each virus sample dilution, the volume pipetted down the side of the well so as not to disturb the VeroE6 monolayer. The plate was incubated for 1 hr at 37°C 5% CO_2_. During this incubation period, a solution comprising a 1:0.7 mixture of cDMEM and 2% methylcellulose (viscosity: 4000 cP) was freshly made and warmed to 37°C in a water bath. After the 1 hr incubation period was over, the supernatant was removed from each well and replaced with 1 mL of the pre-warmed cDMEM:methylcellulose mixture. The culture plate was then returned to the incubator and left undisturbed for 3 days. On the final day, the cDMEM:methylcellulose mixture was removed from each well, cells were fixed with 4% para-formaldehyde in PBS (20 minutes, room temperature), washed with PBS and stained with 0.05% crystal violet (in 20% methanol). After rinsing plates with distilled water, plates were dried, and plaques were counted under a light microscope.

### Histology

The inferior lung lobe was removed from mice and fixed in 10% formalin. Sample processing, paraffin embedding, H&E and acid fast bacilli (AFB) staining was performed by the OSU Comparative Pathology & Mouse Phenotyping Shared Resource (CPMPSR). Immunohistochemistry (IHC) was performed using a monoclonal antibody specific to SARS-CoV-2 Nucleocapsid (clone B46F; ThermoFisher) per previously reported methods [42]. Histology slides were imaged using a Nikon Ti2 widefield microscope fitted with 4x, 10x and 60x CFI Plan Fluor objectives and a DS-Fi3 color camera. Images were processed using FIJI [43] and compiled using BioRender.com. The individual and cumulative areas of each TB granuloma in Mtb^POS^CoV2^NEG^ and Mtb^POS^CoV2^POS^ lungs were determined using our previously reported methods [44].

### ELISA

CoV2 N protein levels in lung homogenates were determined using a commercially available ELISA kit (ADS Biotec), as were protein levels of the cytokines IL1β, IL6 and IFNγ (Biolegend). ELISA kits were used per manufacturer protocols.

### Quantitative Real Time PCR

Lung RNA was extracted from the superior lung lobe using the RNeasy Mini Kit method (Qiagen) and reverse transcribed using the SuperScript VILO cDNA Synthesis Kit method (ThermoFisher). Quantitative real time PCR (qRT-PCR) was performed on a C1000 Touch Thermocycler (Bio-Rad) using SYBR Select Master Mix (Applied Biosystems) per manufacturer protocols. The primer sequences used to amplify cDNA for genes of interest were previously published [45,46]. Each biological replicate was performed in technical duplicate and data were analyzed using the ΔΔCt method.

### Cell purification

To purify live CD45+ cells for single cell RNA sequencing, lungs from uninfected, Mtb- or MACoV2-monoinfected and Mtb/MACoV2 coinfected mice were removed and treated in an identical manner. Lungs were first digested in a DNase/collagenase mixture [47]; dead cells from the resulting slurry were then removed via negative magnetic selection using the Dead Cell Removal kit method (Miltenyi). The live cells were then mixed with CD45 microbeads (Miltenyi) and used for positive magnetic selection of live CD45+ cells. Trypan blue staining was used to confirm cell viability. Cells were the prepared for single cell partitioning via a 10X Genomics Chromium Controller using manufacturer provided protocols (10x Genomics Document Number CG000136). 1 x 10^4^ cells per experimental group were loaded onto the Controller and partitioned, as carried out by the OSU Genomics Shared Resource core.

### Single cell RNA sequencing (scRNA seq)

scRNA-seq libraries were prepared and analyzed using the 10X Genomics and Illumina platforms, respectively, per previously reported methods [48].

### Statistical analysis

All experiments were performed using randomly assigned mice without investigator blinding. All data points and *p* values reflect biological replicates from at least two independent experiments per figure (4 mice per group per timepoint). Statistical analysis was performed using GraphPad Prism. Unpaired, two-tailed Student t tests and one-way ANOVA tests with post hoc Tukey-Kramer corrections were used to assess statistical significance. Graphs were likewise generated in GraphPad Prism. The only exception to this were the t-distributed stochastic neighbor embedding (t-SNE), annotation and graphing associated with our scRNA analysis, which was performed with Cell Ranger and RStudio.

## Supporting information

S1 Fig Lineage defining markers were similarly expressed across uninfected (UI), Mtb^NEG^MACoV2^POS^, Mtb^POS^MACoV2^NEG^ and Mtb^POS^MACoV2^POS^ groups. The distribution and expression patterns of lineage defining genes that were used to annotate each t-SNE cluster, as shown for each individual experimental group (pooled group data are shown in **Fig 5**).
(TIF)
