# Supplementary figures and images for: Mice infected with Mycobacterium tuberculosis are resistant to acute disease caused by secondary infection with SARS-CoV-2
Source: PLoS Pathog. 2022 Mar 24;18(3):e1010093. doi: 10.1371/journal.ppat.1010093 (PMC8946739; doi:10.1371/journal.ppat.1010093)

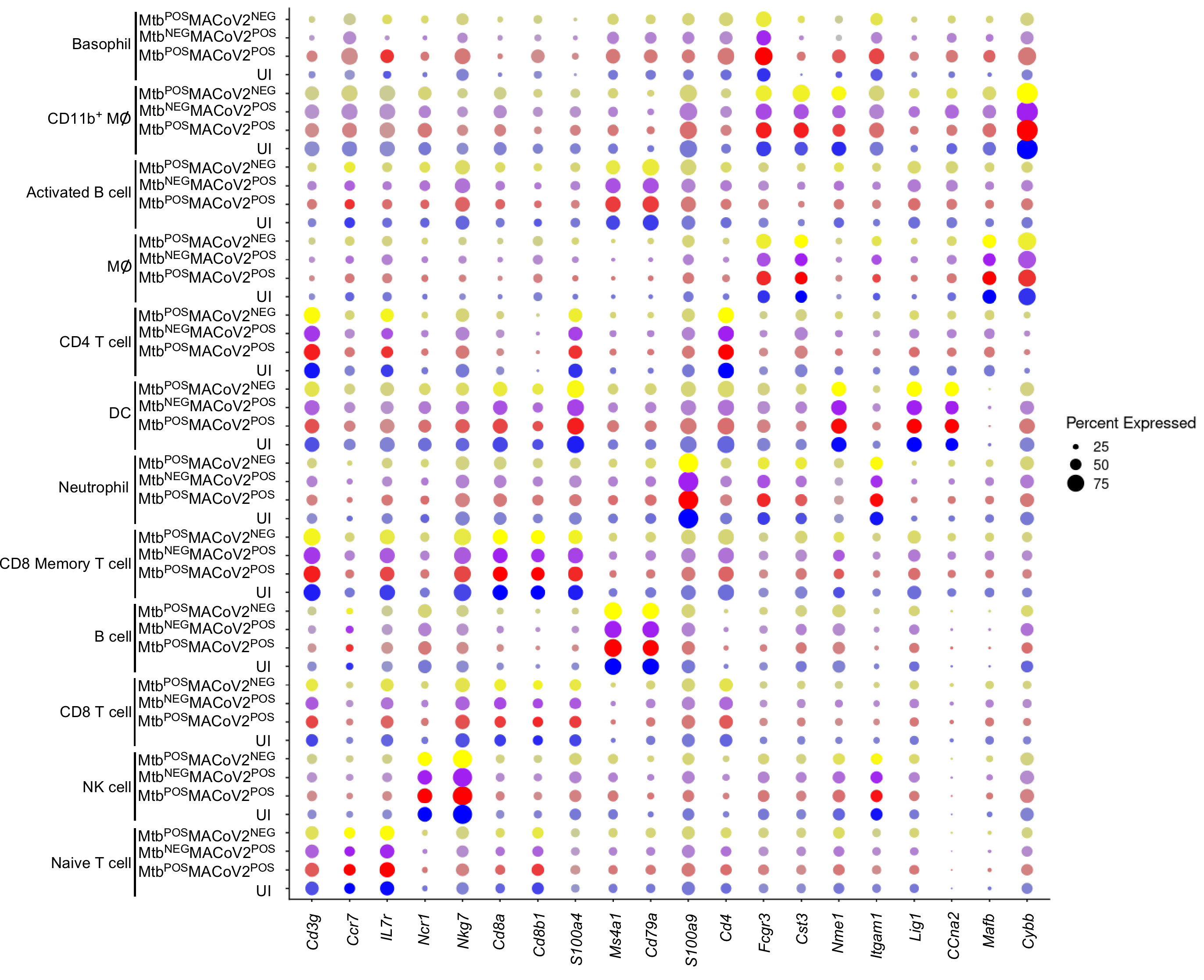

Supplement: S1 Fig — The distribution and expression patterns of lineage defining genes that were used to annotate each t-SNE cluster, as shown for each individual experimental group (pooled group data are shown in Fig 5). (TIF) [file ppat.1010093.s001.tif]
